# Porta hepatic schwannoma: case report and a 30-year review of the literature yielding 15 cases

**DOI:** 10.1186/s12957-016-0858-9

**Published:** 2016-04-02

**Authors:** Sheng-yong Yin, Zheng-long Zhai, Kui-wu Ren, Yun-chuan Yang, Da-long Wan, Xiao-yan Liu, Li-jun Wang, Shu-sen Zheng

**Affiliations:** Department of Hepatobiliary and Pancreatic Surgery, First Affiliated Hospital, Zhejiang University School of Medicine, 310003 Hangzhou, China; Key Laboratory of Combined Multi-organ Transplantation, Ministry of Public Health, Key Laboratory of Organ Transplantation, Division of Hepatobiliary and Pancreatic Surgery, First Affiliated Hospital, School of Medicine, Zhejiang University, Hangzhou, 310003 Zhejiang Province China; Collaborative Innovation Center for Diagnosis and Treatment of Infectious Diseases, First Affiliated Hospital, Zhejiang University School of Medicine, 310003 Hangzhou, China; Department of Pathology, First Affiliated Hospital, Zhejiang University School of Medicine, 310003 Hangzhou, China

**Keywords:** Schwannomas, Hepatoduodenal ligament, Porta hepatis, Proper hepatic artery, S-100

## Abstract

**Background:**

Schwannomas located in the periportal region are extremely rare. Only 14 cases have been reported in the medical literature worldwide. Cases of porta hepatic schwannomas reported in the literature worldwide were reviewed. As a result, it is very challenging for surgeons to make a preoperative diagnosis due to its rarity and nonspecific imaging manifestations.

**Case Presentation:**

A 57-year-old Chinese female was admitted to our institution with complaint of upper abdominal distension and the abdominal CT in the local hospital revealed a hypodense mass in the porta hepatis. A fine needle aspiration (FNA) was made to confirm the diagnosis, but the result was just suggestive of spindle cell neoplasia. Eventually, the patient underwent surgery and postoperative pathology confirmed schwannoma in porta hepatis. The patient recovered uneventfully with no evidence of recurrence after a follow-up period of 41 months.

**Conclusions:**

It is essential for the final diagnosis of porta hepatic schwannomas to combine histological examination with immunohistochemistry after surgery. The main treatment of porta hepatic schwannomas is complete excision with free margins and no lymph node dissection. In some cases, biliary reconstruction or the proper hepatic and the gastroduodenal artery resection was performed because the tumor was inseparably attached to the extrahepatic bile duct or the proper hepatic and the gastroduodenal artery. Malignant transformation of schwannomas is very rare and the overall prognosis is satisfactory.

## Background

The periportal region is a complex anatomic region between the superior aspect of the first portion of the duodenum and the porta hepatis, including the hepatoduodenal ligament and the extrahepatic bile duct, portal vein, hepatic artery, autonomic nerve fibers, and lymph nodes [1]. When patients exhibit symptoms of abdominal pain or jaundice or show no symptoms, and a mass in the periportal region is detected in image scans, the diagnosis of a cholangiocarcinoma or a gastrointestinal stromal tumor (GIST) may be considered first. However, in rare cases, the diagnosis is of a porta hepatic schwannoma. Porta hepatic schwannomas are extremely rare, with only 14 cases reported in the literature to date. Of the 14 cases, the mean age of the patients was 45 years (range 29–74 years), and the male-female ratio was 6:8. The literature depicted tumors varying from 2.2 cm (the average tumor diameter line) to 7.5 cm in size, with a median of approximately 4.8 cm. For the mass in porta hepatic, it is difficult to perform a biopsy because of vessel interposition. So, the surgeons are inclined to make a fine needle aspiration (FNA) because of lower risk of bleeding. In the 14 cases, the FNA was operated only for two patients, and none underwent biopsy before surgery. But, the FNA usually fails to provide the immunohistochemistry, which is imperative to make a diagnosis for the mesenchymal tumors. In most cases, the final diagnosis of schwannoma was confirmed by postoperative pathological examination.

Herein, we report a case of schwannoma located in the porta hepatis and review cases reported in the literature worldwide for a better understanding of the clinical and pathological features of porta hepatic schwannomas.

## Case presentation

**Fig. 1:**
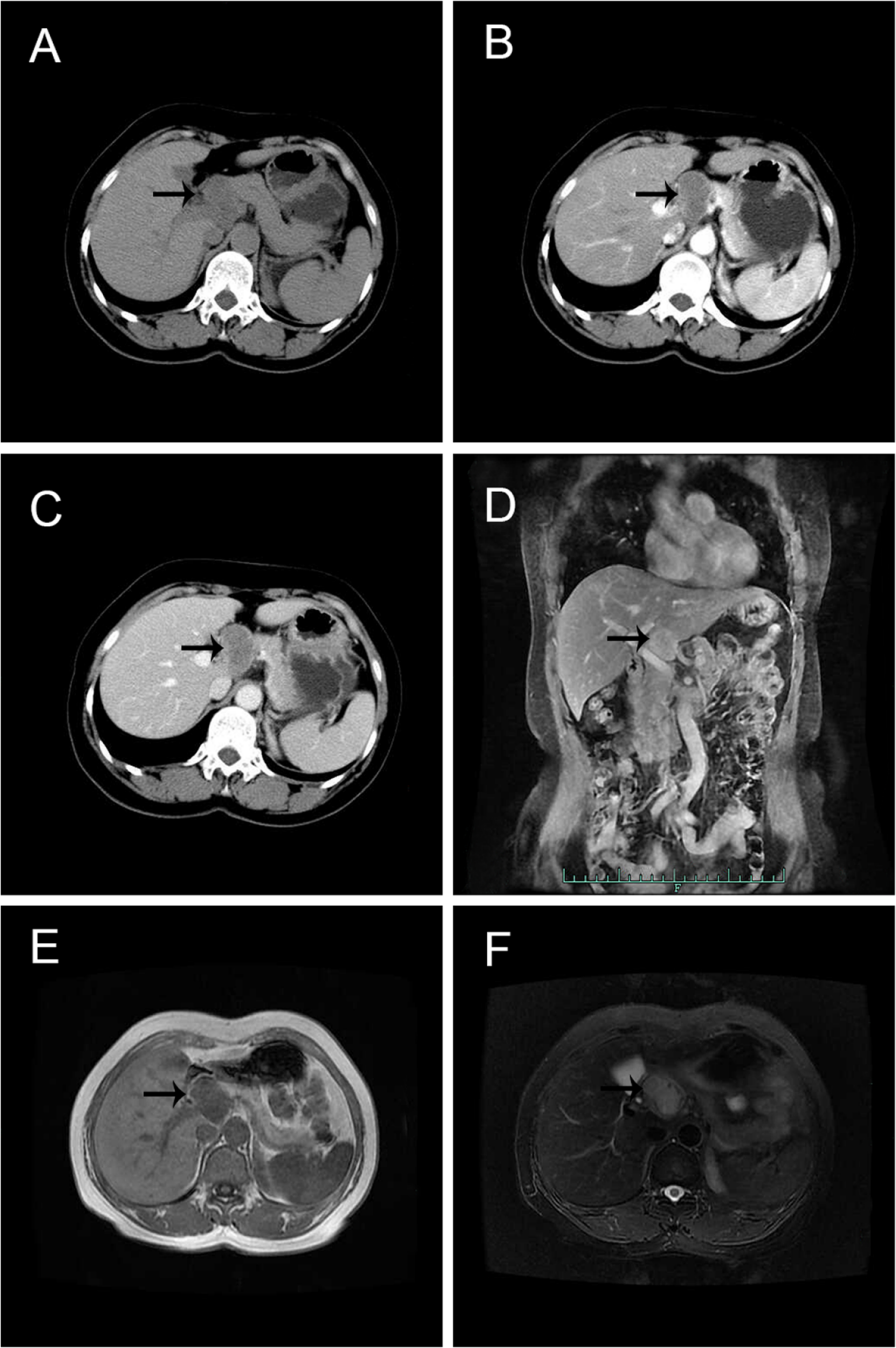
Abdominal CT and MRI findings of the patient. **a** Plain CT scan revealed a 4.0 × 3.2 × 3.0-cm hypodense mass in the porta hepatis (*black arrow*). **b** Enhanced CT scan revealed a mildly enhanced mass in arterial phase (*black arrow*). **c** Moderately enhanced mass in portal phase (*black arrow*). **d** MRI revealed a 4.0 × 3.0-cm mass in the sagittal section (*black arrow*). **e** Low signal intensity in T1-weighted imaging (*black arrow*). **f** High signal intensity in T2-weighted imaging (*black arrow*)

**Fig. 2:**
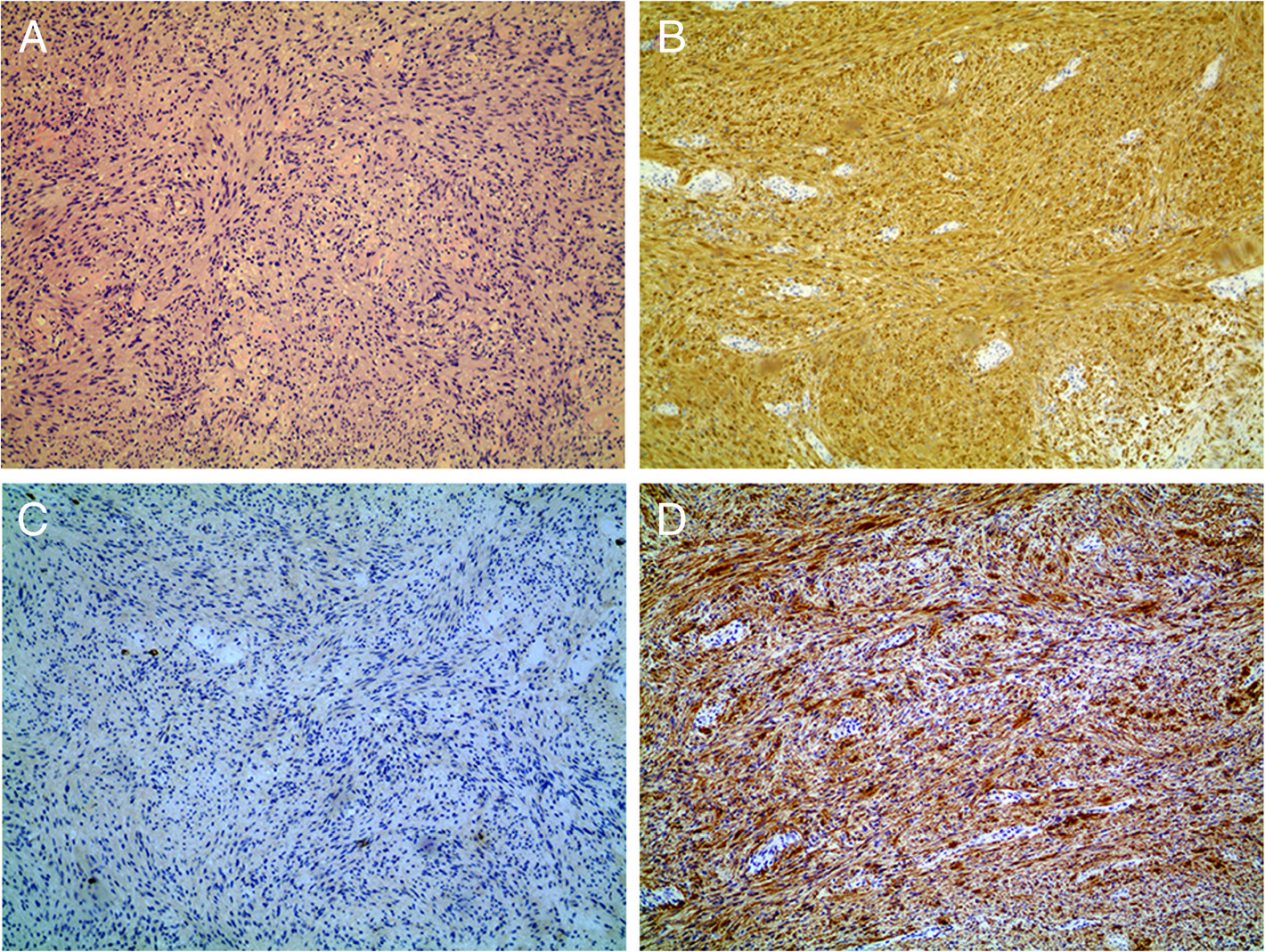
**a** Histological examination findings of the tumor: bundles of uniform spindle cells whose elongated nuclei were arranged in a palisading pattern (hematoxylin and eosin; magnification, ×100). **b** Immunohistochemistry revealed that tumor cells were positive for S-100 protein (magnification, ×100). **c** Immunohistochemistry revealed that tumor cells were negative for CD117 (magnification, ×100). **d** Immunohistochemistry revealed that tumor cells were positive for GFAP (magnification, ×100)

On October 12, 2011, a 57-year-old Chinese female was admitted to our institution with complaint of upper abdominal distension for 2 weeks. She denied any abdominal trauma or surgeries in her life. No specific findings were noted in her family history and personal history. And, she was taking no medications. Before this treatment, she went to the local hospital where an abdominal ultrasound was performed revealing a mass in the head of the pancreas. No abnormalities were detected upon clinical examination and in laboratory investigations. The computer tomography (CT) scan (Fig. 1a–c) revealed a 4.0 × 3.2 × 3.0-cm hypodense mass in the porta hepatis, mildly enhanced in arterial phase, and moderately enhanced in portal phase. There was no clear boundary between the mass and the caudate lobe of the liver, and the lower edge of the lesion was close to the neck of pancreas, pressing down on the common hepatic artery. No enlarged hilar lymph nodes or exogenous caudate lobe tumor were identified. The magnetic resonance imaging (MRI) (Fig. 1d–f) showed the long T1 and T2 signal mass in the porta hepatis, with 4.0 × 3.0 cm. The imaging feature on MRI was similar to CT. Fine needle aspiration guided by endoscopic ultrasonography revealed spindle cells arranged in bundles and palisades, with no nuclear atypia, suggestive of a spindle cell neoplasia. With a high suspicion of a GIST, diagnostic laparoscopy was performed. The tumor was located in the porta hepatis, tightly pressing the hepatoduodenal ligament, and the caudate lobe and the pancreas were not involved. Then, after careful dissection, the tumor was found to be in close contact with the common hepatic artery and cystic artery. Subsequently, the patient underwent laparotomy, and intraoperative frozen-section examination revealed a mesenchymal tumor in the porta hepatis, highly suspicious of a GIST. So, the tumor was macroscopically resected en-bloc with the invaded common hepatic artery, and the blood supply of the proper hepatic artery was good through the gastroduodenal artery. And, cholecystectomy was also performed. Post-operative detailed histopathological examination showed benign schwannoma (Fig. 2a–d): CD117(−), S-100(+), GFAP(+), CD34(−), SMA(−), and Ki-67(+, little). Postoperatively, the patient recovered uneventfully. No symptoms or signs of recurrence have been observed in our patient during the 41 months of follow-up.

### Literature review

PubMed, Google scholar (http://scholar.google.com.cn/), the National Knowledge Infrastructure (http://www.cnki.net//), the Chinese periodical Database of Science and Technology (http://lib.cqvip.com/), and the Wanfang Data Knowledge Service Platform (http://www.wanfangdata.com.cn/) were searched for cases of porta hepatic schwannoma between 1985 and 2015, adding up to 14 patients. Details of all the 14 cases and the current case shown in Tables 1 and 2 summarized the important available clinicopathological factors. Continuous data are presented as mean ± standard deviation and range.

**Table 1:** Summary of cases of porta hepatic schwannoma

Author	Year	Sex/age	Presenting symptoms	Imaging examination	Size (cm)	Location	Primary diagnosis	Treatment	Follow-up (months)
Nagafuchi [17]	1993	F /62	Asymptomatic	US + CT + MRI + ERC + CA	6.2	Hepatoduodenal ligament	Acute hepatitis and hilar mass	SR	26 (no relapse)
Fang [24]	1995	F /33	Abdominal distension + jaundice	US	4.5	Porta hepatis	Portal hypertension and hilar mass	SR	NO
Huang [25]	1996	M/41	Abdominal pain	US + CT + IVP + GIBI	7.2	Hepatoduodenal ligament	Abdominal mass	SR	NO
Choi [26]	2001	M/37	Asymptomatic	US + CT + MRI	5	Porta hepatis	Hilar mass	SR	NO
Park [27]	2006	F/53	Asymptomatic	CT	4.5	Porta hepatis	Hilar mass	SR	11 (no relapse)
Wang [28]	2006	M/45	Abdominal distension + nausea	US + CT + MRI + MRCP	7	Porta hepatis	Klatskin’s tumor	SR	NO
Zhang [29]	2009	F/42	Asymptomatic	US + CT	2.2	Hepatoduodenal ligament	Left hepatic lobe mass (liver cancer not excluded)	SR	NO
Kulkarni [4]	2009	M/38	Abdominal pain + jaundice + weight loss	CT	4.5	Porta hepatis	Hilar mass	SR	3 (no relapse)
Li [12]	2010	F/38	Abdominal distension	US + CEUS + CT	4	Porta hepatis	FNH considered (FNA)	SR	NO
Pinto [30]	2011	M/29	Asymptomatic	US + EUS + MRI	3.7	Hepatoduodenal ligament	Spindle cell neoplasia or stromal tumor (FNA)	SR	NO
Huang [22]	2011	F/45	Abdominal pain	CT	7.5	Proper hepatic artery	Hilar mass	SR	36 (no relapse)
Present case	2011	F/57	Abdominal discomfort	US + CT + MRI + EUS	3.5	Porta hepatis	Spindle cell neoplasia (FNA)	SR	41 (no relapse)
Wang [31]	2012	M/62	Abdominal pain	US + CT + MRI	3.5	Porta hepatis	Hilar mass (probably liver abscess)	SR	4 (no relapse)
Wang [32]	2012	F/74	Asymptomatic	CT + MRI + MRCP	2.5	Porta hepatis	Hilar mass	SR	NO
Chen [18]	2015	F/69	Abdominal distension	CT	4.7	Hepatoduodenal ligament	GLNH (plasma cell type) considered	SR	NO

*M* male, *F* female, *NA* not available, *CEUS* contrast-enhanced ultrasound, *EUS* endoscopic ultrasonography, *ERC* endoscopic retrograde cholangiogram, *CA* celiac angiography, *GIBI* gastrointestinal barium imaging, *IVP* intravenous pyelography, *FNH* focal nodular hyperplasia, *FNA* fine needle aspiration, *SR* surgical resection, *GLNH* giant lymph node hyperplasia

**Table 2:** Summary of clinicopathological data from 15 cases of porta hepatic schwannoma

	*n* (%) or mean ± SD (range)
Age (years)	48.3 ± 13.7 (29–74)
Sex (male/female), (male %)	6/9 (40 %)
Symptoms	
Asymptomatic	6 (40 %)
Symptomatic	
Abdominal distension/abdominal discomfort	5 (33 %)
Abdominal pain	4 (27 %)
Jaundice/dark urine/colorless stool/pruritus	2 (13 %)
Weight loss	1 (7 %)
Nausea/vomiting/anorexia	1 (7 %)
Location	
Porta hepatis	9 (60 %)
Hepatoduodenal ligament	5 (33 %)
Proper hepatic artery	1 (7 %)
Mean size (cm), (*n* = 15)	4.7 ± 1.6 (2.2–7.5)
Mean follow-up months (*n* = 6)	16.0 ± 14.5 (3–41)
Disease related mortality	0 (0 %)

### Discussion

Schwannoma, also known as neurilemmoma, is a tumor derived from Schwann cells, which form the inner portion of the peripheral nerve sheath [2]. The exact etiology and pathogenesis of schwannomas remain unclear, but recent research has indicated that defects in merlin gene are responsible for both sporadic and genetically acquired schwannomas, and the mechanisms by which merlin loss triggers tumor development are being unraveled [3]. Theoretically, the tumor can affect any nerve trunk or any organ, except the olfactory and optic nerves, which lack Schwann cells [4]. The most common locations for schwannomas are the upper extremities, trunk, head, and neck, retroperitoneum, mediastinum, pelvis, and peritoneum [5]. Intraperitoneal schwannomas are relatively rare and mostly located in solid organs such as the liver and the pancreas. However, porta hepatic schwannomas are extremely rare, with only 14 cases reported in the literature to date. The sympathetic and parasympathetic fibers are distributed along the hepatic and gastroduodenal arteries, with their branches interwoven into a network, which is the anatomical location of occurrence of the porta hepatic schwannoma [6].

Schwannomas most commonly occur in patients between the ages of 20 and 50 years with equal frequency in men and women [7]. For cases of porta hepatic schwannoma, the mean age of the patients was 48 years (range 29–74 years), and the male-female ratio was 6:9. Symptoms caused by schwannomas vary with the site and size of the tumors. In the 15 cases, the lesion was located in the porta hepatis in nine patients (60 %), hepatoduodenal ligament in five patients (33 %), and proper hepatic artery in one patient (7 %). The literature depicts tumors varying from 2.2 cm (the average tumor diameter line) to 7.5 cm in size, with a median of approximately 4.7 cm. Patients with porta hepatic schwannomas may exhibit symptoms by compressing adjacent structures such as the bile duct and the gastrointestinal tract. In the literature, 40 % of the patients were asymptomatic and 60 % of the patients were symptomatic. The most common symptom was abdominal distension/abdominal discomfort (5/15 patients or 33 %), followed by abdominal pain (4/15 patients or 27 %), jaundice/dark urine/colorless stool/pruritus (2/15 patients or 13 %), nausea/vomiting/anorexia (1/15 patients or 7 %), and weight loss (1/15 patients or 7 %).

Patients with porta hepatic schwannomas usually present with a porta hepatis or hepatoduodenal ligament mass on imaging studies, and it is very challenging for surgeons to make a preoperative diagnosis due to its rarity and nonspecific clinical and imaging characteristics. The ultrasound imaging often shows isoechoic or hypoechoic solid masses with well-defined limits. Generally, a CT scan shows a well-defined hypodense heterogenous mass with peripheral enhancement [8]. Schwannomas on the MRI are usually masses with hypointensity on T1-weighted images and heterogenous hyperintensity or, sometimes internal heterogeneous mixed hypo and hyperintensity on T2-weighted images [9]. Author Cohen has presented histologic evidence suggesting that the hypo-density areas and inhomogeneities in schwannomas are due to hypocellular areas adjacent to more cellular regions and/or hypocellular areas adjacent to dense bundles of collagen, xanthomatous formation, and cystic degeneration within the tumors [10]. In the present case, the CT and MRI showed a homogeneous mass with peripheral enhancement in the porta hepatis. When a solitary, well-demarcated mass occurs in the porta hepatis, differential diagnosis should be made including the following diseases: lymphoma, giant lymph node hyperplasia (GLNH), lymph node tuberculosis, and lesser sac benign smooth muscle tumors such as leiomyomas, nerve sheath tumors, and hemangiomas originating in the lesser sac as well as stromal tumors of the gastrointestinal tract protruding into the lesser sac [11]. Considering that imaging manifestations of porta hepatic schwannomas are nonspecific, patients are usually misdiagnosed preoperatively, unless by biopsy. In the 15 cases, the FNA was operated only for three patients, including the present case. However, the result of FNA may be inconsistent with final pathologic results [12]. Therefore, postoperative pathologic examination is still the gold standard in the diagnosis of schwannoma.

Overall, it is essential for the diagnosis of schwannoma to combine histological examination with immunohistochemistry. Macroscopically, small schwannomas tend to be spheroidal, whereas larger tumors can be sausage-shaped, ovoid, or irregularly lobulated [13]. Larger schwannomas have a tendency to undergo secondary degeneration such as pseudocystic regression, calcification, and hemorrhage [14], which can explain inhomogeneities in a portion of schwannomas. In the 15 cases, seven cases (47 %) underwent cystic changes or calcification. Microscopically, the hallmark of schwannoma is the pattern of alternating Antoni A and B areas, with varying relative amounts [15]. The Antoni A area is hypercellular and characterized by closely packed spindle cells with occasional nuclear palisading and Verocay bodies, whereas the Antoni B area is hypocellular and is occupied by loosely arranged tumor cells [16]. Two kinds of structures often coexist in the same tumor, but most with one main type. There were two cases of cellular schwannoma in 15 cases characterized by predominantly Antoni A areas histologically [17, 18].

Immunohistochemical staining is strongly and diffusely positive for S-100 protein in a schwannoma consistent with the finding of a nerve sheath tumor [10]. A few gastrointestinal stromal tumors are positive for S-100; however, they are also positive for either CD34 or CD117 [19]. As a contrast, a schwannoma is negative for both CD34 and CD117 [20]. A leiomyoma would be negative for S-100 and positive for desmin or smooth muscle actin [19]. In addition, we also demand to make a differential diagnosis with malignant peripheral nerve sheath tumor (MPNST), although malignant transformation of these tumors is very rare [21]. MPNSTs are usually characterized by high cell density, obvious cell atypia, more pathological mitotic, diverse cellular components, and it is common to see tumor necrosis, CK(+), EMA(+), and CEA(+) [18].

The main treatment of porta hepatic schwannomas is complete excision with free margins and no lymph node dissection. In the 15 cases, seven patients underwent complete tumor excision. But, two patients underwent tumor excision en bloc with hepatic artery resection because of the intraoperative finding of tumor invasion of the hepatic artery (including the present case) [22]. In addition, one patient underwent tumor or bile duct excision and Roux-en-Y hepaticojejunostomy because the tumor was attached to the extrahepatic bile duct [4]. One patient underwent tumor excision en bloc with T-tube drainage because a small rupture of the bile duct wall caused bile leakage during the dissection of the extrahepatic bile duct [17]. One patient underwent tumor excision en bloc with right hepatectomy because the right anterior portal vein was invaded [12], and the details of surgical resection were not specified in three patients. Postoperative pathological results indicated 13 cases of benign schwannoma and two cases of cellular schwannoma which is considered as a subtype of schwannoma [17, 18]. The complete excision of the tumor is curative and most cases do not relapse and additional treatments are not necessary. The overall prognosis is very good [23]. In the follow-up of 6/15 patients, there was no recurrence with a mean follow-up of 16 months (range 3–41 months).

## Conclusions

Porta hepatic schwannomas are extremely rare. It is a big challenge to make a correct diagnosis preoperatively due to its rarity and nonspecific imaging and clinical manifestations. Postoperative pathologic examination is still the gold standard in the diagnosis of schwannoma. The main treatment of benign schwannomas is complete excision with free margins and no lymph node dissection and biliary surgery when needed. Malignant transformation of schwannomas is very rare and the overall prognosis is satisfactory after surgery.

## Consent

Written informed consent was obtained from the patient for publication of this Case report and any accompanying images. A copy of the written consent is available for review by the Editor-in-Chief of this journal.
